# Mobility-Enhanced Reliable Geographical Forwarding in Cognitive Radio Sensor Networks

**DOI:** 10.3390/s16020172

**Published:** 2016-01-29

**Authors:** Suleiman Zubair, Sharifah Kamilah Syed Yusoff, Norsheila Fisal

**Affiliations:** UTM-MIMOS Centre of Excellence in Telecommunication Technology, Faculty of Electrical Engineering, Universiti Teknologi Malaysia, 81310 UTM Johor Bahru, Malaysia; kamilah@fke.utm.my (S.K.S.Y.); norsheila@utm.my (N.F.)

**Keywords:** mobility, cognitive radio *ad hoc* wireless networks, cognitive radio sensor network, geographical forwarding, speed, reliable routing, opportunistic routing, routing protocol, virtual clustering

## Abstract

The emergence of the Internet of Things and the proliferation of mobile wireless devices has brought the area of mobile cognitive radio sensor networks (MCRSN) to the research spot light. Notwithstanding the potentials of CRSNs in terms of opportunistic channel usage for bursty traffic, the effect of the mobility of resource-constrained nodes to route stability, mobility-induced spatio-temporal spectral opportunities and primary user (PU) protection still remain open issues that need to be jointly addressed. To this effect, this paper proposes a mobile reliable geographical forwarding routing (MROR) protocol. MROR provides a robust mobile framework for geographical forwarding that is based on a mobility-induced channel availability model. It presents a comprehensive routing strategy that considers PU activity (to take care of routes that have to be built through PU coverage), PU signal protection (by the introduction of a mobility-induced guard (mguard) distance) and the random mobility-induced spatio-temporal spectrum opportunities (for enhancement of throughput). It also addresses the issue of frequent route maintenance that arises when speeds of the mobile nodes are considered as a routing metric. As a result, simulation has shown the ability of MROR to reduce the route failure rate by about 65% as against other schemes. In addition, further results show that MROR can improve both the throughput and goodput at the sink in an energy-efficient manner that is required in CRSNs as against compared works.

## 1. Introduction

The wide proliferation of mobile wireless devices and applications is introducing new research challenges in cognitive radio networks. According to the latest worldwide study, for industrial automation alone, the installed base of wireless Internet of Things (IoT) devices reached 10.3 million in 2014 [1]. Furthermore, it is globally recorded that smart devices represented 26 percent of the total mobile devices and connections in 2014; they accounted for 88 percent of the mobile data traffic [2]. Thus, in addition to the spectrum management issues that arise from this trend [3,4], the fact that the huge emerging network is basically characterized by sensors has brought the area of mobile cognitive radio sensor networks (MCRSN) to the research spot light [5].

Mobility is among the most important factors that affect the effective communication in wireless systems. Although it affects all of the communication layers, however, it poses unique challenges to the routing layer, because it affects numerous network characteristics, such as network capacity [6], connectivity [7] and coverage [8]. It is also an inherent feature to support various types of wireless services in cognitive radio sensor networks (CRSNs). Despite its importance, however, mobility has not been adequately addressed in the context of resource-constrained routing in dynamic spectrum access [5]. As at this time, no work has been proposed to tackle the issue of mobile routing in CRSNs. Although, there exist some proposals in the realm of cognitive radio *ad hoc* networks (CRAHNs), which can seemingly be assumed to be adaptable for CRSNs. However, this is not the case because of the unique resource constraint characteristics of CRSNs, specifically in terms of computation, energy and memory capacity [3]. Nevertheless, the issue of mobile routing in CRAHNs is still an open research issue, as most proposed routing metrics mainly considered metrics that do not directly reflect the effect of node mobility [5]. The main issues that such proposed metrics failed to address with respect to mobility include high route instability and mobile estimation of channel availability. These two metrics are the direct implication of introducing mobility into CR networks. Thus, the effectiveness of any protocol will be seriously impacted if both are not considered as a routing metric. As these metrics are still yet to be incorporatedas active routing metrics [5], the motivation for this work (as further elucidated in Section 2) is mainly to propose a routing scheme that incorporatesboth metrics in routing for CRSNs.

It should be recalled that, the CRSN paradigm was introduced to improve overall spectrum utilization. Thus, by dynamically changing its operating parameters, CRSN nodes (the Secondary Users, SUs), senses the spectrum, determines the vacant bands, and makes use of these available bands in an opportunistic manner. With these capabilities, CRSN nodes can operate in both licensed and unlicensed bands. In licensed bands, wireless users with a specific license to communicate over the allocated band (the primary users, PUs), have the priority to access the channel. SUs, are only allowed access to the channel with the condition of not causing interference to the PUs. When a PU starts communication, SUs must detect other potentially vacant bands (spectrum sensing), decide on which channel move to (spectrum decision), and then finally adapt its transceiver so that the active communication is continued over the new channel (spectrum handoff). This sequence of operation which outlines a typical cognitive cycle can also be applied by the SUs over an unlicensed band.

This work proposes a mobility-enhanced reliable opportunistic routing (MROR) scheme for routing in CRSN. MROR provides a robust mobility-enhanced framework that is based on a mobility channel availability model, which is still yet to be considered in mobile CRSNs. It proposes a comprehensive routing strategy that considers PU activity (to take care of routes that have to be built through PU coverage), PU signal protection (by the introduction of a mobility-induced guard (mguard) distance) and the random mobility-induced spatio-temporal spectrum opportunities (for enhancement of throughput). It also addresses the issue of frequent route maintenance that arises when speed of the mobile nodes are considered as a routing metric. In this respect, MROR will be the first that considers the speed of mobile nodes in the creation of routing paths. In addition, it also addresses the “weak links” phenomena by considering the link quality of communicating nodes before data forwarding. This was addressed by disconnecting between the assignment of the channel and the next hop process via the formation of virtual clusters. This approach is contrary to the prevalent routing approach usually adopted for CR based *ad hoc* networks [9]. In MROR, the control channel (CC) is utilized for control signaling, while searching for all possible routes (between source and sink nodes) is handled by the route request phase. Based on the developed mobility-based bandwidth estimation and delay, the sink is made to select the best route that supports the required quality of service (QoS) level. In the dynamic reactive forwarding process of MROR, initially, all nodes along the chosen path are referred to as local minimum resolution (LMR) nodes. Then, by introducing a virtual mobile handover (VMH) zoning system, these roles are seamlessly transferred to eligible mobile nodes as long as the path remains active. Based on the correlation of spectrum opportunities, the route reply process is used to group the neighbors of each LMR node into a virtual contention (VC) group. The data forwarding phase is handled by the VC group (VCG) receiver-based contention forwarding scheme. A VCG refers to the grouping of nodes around an LMR based on available spectral coloration. This scheme is used for reduction of media access contention among participating nodes, which in effect increases the chance of successful data transfer across the VCGs to the sink. Simulation results show how the MROR framework effectively utilizes CR capabilities to improve communication in MCRSN and seamlessly manage the mobility factor to greatly reduce frequent route maintenance activities when compared to similar solutions.

In the implementation of a reliable and efficient mobility-enhanced geographical forwarding scheme for MCRSNs, the major contributions this work achieves can be outlined as follows: (i) the mobility-induced route instability in MCRSNs is resolved by incorporating a channel availability model into the routing metrics that reflects the fluctuation of mobility-induced spectrum opportunities; (ii) the scheme provides a framework for boosting throughput in MCRSNs, by compensating the degradation in the sensitivity of energy detection by the spatial-temporal diversity of the mobile nodes; (iii) in order to ensure primary user (PU) signal protection, the scheme introduces the use of a mobility-induced guard (mguard) distance, which is jointly considered with the random mobility, induced spatio-temporal spectrum opportunities and the PU activity (to take care of routes that have to be built through PU coverage). In this regard, MROR will be the first to implement mobility-enhanced, lossy link-aware geographic forwarding for MCRSNs.

This paper is organized as follows: The motivation for this work is discussed in Section 2. In Section 3, a review of relevant and related works on routing in CRSN is discussed. In Section 4, the mobile channel availability model is presented. Section 5 the features, the detailed functions and the operation of the modules that make up MROR are discussed. In Section 6, the results for the performance evaluation are discussed. Section 7 presents a comparative analysis of MROR with related works. Section 8 concludes the paper.

## 2. Motivation

This section discuses the motivation behind this work, which is fundamentally centered around high route instability, channel availability estimation and protection of the PU signal.

High route instability: Unpredictable and fast movements of secondary users (SUs) make guaranteeing QoS requirements and minimizing interference to PUs more challenging. This is because the higher mobility of SUs can reduce channel access time and, hence, increase the number of channel switches [5,6,10]. This in turn incurs a higher cost in terms of energy and delay because of the frequent initiation of route maintenance or rerouting activities. Most routing protocols have bypassed this issue by either not mentioning the considered speed of the SUs [11] or pegging the movement of the nodes to very low limits [12], which have no significant difference from static scenarios because of the high tendency of spectrum correlation at such a speed [13]. In other cases, researchers take the choice of always creating routes that bypass the coverage area of the primary user (PU) [11,14]. However, this may not always be the ideal case, because network traffic in some cases has to go through the coverage area of the PU, especially in the case of sensor networks. Since a target area can be located anywhere. At this point, it is important to mention that the fact that reactive routing generally suits mobile scenarios [15] does not make them altogether suitable for CRSNs. This is because in addition to mobility, the dynamic spectrum access component of CRSNs, which incorporates spectrum sensing and channel switching, further amplifies the issue of route instability. Thus, this still remains an open area that needs to be addressed in CRSNs.

Estimation of channel availability: With respect to this metric, the majority of routing protocols in CRAHNs can be generally classified into two categories. First are protocols that derive the spectrum-availability model based solely on PUs’ temporal traffic statistics [12] without considering the benefits of spatial-temporal diversity of PU signals that comes with mobility. This greatly limits the potential of the network, especially when bursty applications are considered. Second are protocols that have assumed the perfect knowledge of PU activity for channel estimation [11]. In this case, the protocol does not practically consider the dynamics of this metric in the routing decision. Both works [11,12] also assume that cooperative sensing is used, while, in reality, implementing meaningful cooperative sensing in mobile scenarios is very challenging and adds more overhead, which are not trivial, especially in the realms of CRSNs. With energy detection, it has been shown that spatial-temporal diversity in the received PU signals for a single mobile CR user increases with mobility [16]. In addition, it was also shown that, without cooperative detection, the mobility of the CR node improves PU detection performance, because as speed increases, the observations are less correlated. Consequently, this property reduces the frequency of periodic sensing, which means more time slots for active data transfer by the CR user. However, it is important to mention that mobility can also reduce the average received signal strength. This calls for the need to compensate the degradation in the sensitivity of energy detection by the spatial-temporal diversity [16].

Primary user protection: In order to ensure protection of the PU communication, routes are usually built to bypass the PU coverage area. Thus, the considered metrics are typically the PU’s ON and OFF times and the location of the node with respect to the PU coverage area. However, this is not usually the case when CRSNs are considered. This is because an event area that is supposed to be monitored can lie within the PU coverage area. Thus, building routes for mobile CRSNs must jointly consider the PU’s activity, PU signal protection and the random spatio-temporal spectrum opportunities that are mobility induced. This is still a major open issue, which this work seeks to address.

In addition, experimental studies [17,18] have shown the importance of considering the highly unreliable nature of wireless links when considering higher layer protocols. Although this work also considers the issue of instant channel quality assessment as a fundamental component, the details regarding this aspect will only be discussed where necessary. Thus, in order to keep this paper concise, we refer readers to our previous work *reliable geographical forwarding in cognitive radio sensor networks using virtual clusters*(ROR) [13] for the detailed fundamentals of implementing instant channel quality assessment for CRSNs.

In line with the above-mentioned issues, implementing mobile routing can be more challenging because of the unique resource-constrained nature of CRSNs. In [13], we noted that two specific reasons make geographic forwarding schemes the best choice for routing in CRSN: (i) the minimal stored state; this is because, in order to forward packets, the transmitting node needs to only store location information of its immediate neighbors; (ii) high energy and bandwidth conservation; this is because the discovery floods and state propagation are not required beyond a single hop. In [13], we proposed a novel geographical forwarding technique that does not restrict the choice for next hop selection to the nodes in the previously-selected route. ROR is characterized by the seamless formation of virtual clusters and the utilization of instantaneous link quality as the criteria for the next hop selection. Furthermore, ROR ensures network stability by utilizing receiver contention prioritization and idle listening to avoid the occurrence of routing hot spots and to minimize network energy. When compared to the usual CRHANrouting strategy of joint channel and next hop selection, it was proven that ROR makes more per-hop advancement to the sink, while ensuring channel quality. However, the design of ROR did not consider the high route instability and mobile estimation of channel availability, which reflects the fluctuating spectrum opportunities that are induced by SU mobility. Thus, in this work, we propose another version of ROR, which is specifically modeled considering high route instability and mobile estimation of channel availability. This version of ROR, which is called MROR, builds on the strengths of ROR to properly address routing in mobile CRSNs.

## 3. Related Work

One of the most relevant works in this regard is the spectrum-aware routing protocol for cognitive *ad hoc* networks (SEARCH) [12]. SEARCH is a geographic forwarding-based scheme, which jointly undertakes path and channel selection to avoid regions of PU activity during route formation. During the route operation, it can adapt to the newly-discovered and lost spectrum opportunity while considering node mobility in a distributed environment. The key functionality of the SEARCH approach is its evaluation to decide on when the coverage region of the PU should be circumvented and when changing the channel is a preferred option. Although SEARCH is also a geographical forwarding scheme as MROR, MROR implements a lossy link-aware geographic forwarding for CRSNs, which is a more realistic consideration. The MROR approach to routing is more energy efficient and more scalable than the SEARCH approach in the following ways: (i) in SEARCH, route search is broadcast via all channels in order to identify all possible routes to the sink. Thus, the authors did not consider energy, which is a fundamental routing metric in resource-constrained CRSNs [3]. Contrarily, MROR uses the concept of the common control channel (CCC). The design of a simple CCC acquisition scheme for CRSNs has also been proposed by the same authors [19]; (ii) In MROR, the data-forwarding phase is more stateless and scalable, as the next hop is not restricted to a single node as in SEARCH; rather, the next hop is instantaneously decided upon based on the best node that meets the required QoS criteria; (iii) Fundamentally, SEARCH restricts the movement of the nodes to low movement, which has no significant difference from static scenarios because of the high tendency of spectrum correlation at the considered speed. Furthermore, its routes are built to always bypass PU coverage with the aid of anchor locations, which are out of PU coverage. This is the main reason why speed was not considered as a routing metric. As already mentioned, MCRSNs have to consider all possibilities of having routes both within and outside the PU coverage area. Thus, MROR considers a mobility channel availability model as a fundamental routing metric that protects the PU signal within the PU coverage area by considering the PU’s activity, but also increases the routes throughput by seizing the spatio-temporal spectrum opportunities that are mobility induced.

Similar to SEARCH, the geographic routing protocol for cognitive radio mobile *ad hoc* networks (TIGHT) [14], is also built to completely bypass PU activity areas. However, unlike in SEARCH, TIGHT proposes three routing modes. The greedy mode in TIGHT utilizes greedy geographic forwarding to route a packet until it encounters a PU region, after which it routes the packet around the PU region to where greedy forwarding can take over. The other two modes that work best when PUs are active most of the time include the optimal mode, which routes a packet along an optimal trajectory to the sink, and the suboptimal mode, which routes it along a suboptimal trajectory. However, unlike SEARCH and MROR, TIGHT considers only a single PU channel scenario, which is a simpler case to handle. This is because the multi-channel issue, which is a major characteristic of CR networks, is easily bypassed by making all nodes tune to the only PU channel available by default. In addition, just as in SEARCH, by always avoiding PU active areas, it bypasses the mobility-induced spectrum opportunity fluctuation. Furthermore, it does not address the issue of route stability with respect to the speed of the mobile node.

In [11], a distributed cognitive radio routing protocol for *ad hoc* networks (CRP) was proposed. The main difference CRP has in contrast to SEARCH is, in addition to bypassing the PU activity area, CRP also supports the on-demand creation of multiple classes of routes based on service differentiation in CR networks. Although these service-differentiated routes could pass through the PU activity area, the routing metric did not consider spectrum opportunity fluctuation due to mobility, nor did it consider the effect of mobility on the stability of the built routes. In order to reduce the computational overhead at the destination, CRP uses CCC for signaling purposes. On the contrary, MROR uses the spatio-temporal opportunities that are induced by mobility to compensate the reduced channel access opportunity for routes that have to go through PU coverage. While for routes that are built out of the PU coverage, it considers a mobility-induced guard (mguard) distance in addition to the keep-out distance, which ensures adequate primary transmission protection based on the interference temperature limit (ITL) and significantly improves the throughput thereof.

The mobility-assisted routing algorithm with spectrum awareness (MARSA) [20] was proposed to select relays based on both the probability that a node meets the destination and the chance of the existence of at least one available channel when they meet. MARSA, which was mainly motivated by analyzing the real-world phenomenon and trace data of human mobility habits, is based on the deduced rule that the approximately regular PU behaviors result in the approximate regularity of the mobility of the spectrum that could be available for SUs. Hence, it basically identifies a set of channels according to location and sets them as home channels. A central server is used to always update nodes about this channel. When a node comes into the area, it automatically switches to the available home channel for communication. Otherwise, it stores the information until it enters a location where a free home channel exists; only then will it transmit its data.

**Table 1:** Notations. PU, primary user; SU, secondary user.

Symbol	Definition
$\lambda_{su}$	SU reception threshold
$\lambda_{pu}$	PU reception threshold
*S*	SU received signal
$H_{0}$	PU’s hypothetical idle state
$H_{1}$	PU’s hypothetical active state
n(t)	additive white Gaussian noise (AWGN)
$P_{d}$	probability of detecting PU activity
$P_{f}$	probability of false detection of PU activity
$S^{p}\left(t\right)$	PU signal waveform
$\lambda_{pu}$	spectrum sensing decision threshold
*n*	number of SUs
$P_{o}$	SU’s transmission power
$d_{o}^{}$	SU’s transmission reference distance
*α*	path-loss exponential
$Z_{o}$	PU’s transmission radius
$Z_{e,i}$	base station’s keep-out radius
$I_{i}$	Interference on channel *i*
$\ell_{i}$	mobility-induced guard (mguard) distance
$P_{i}$	PU transmission power on channel *i*
ℜ	transmission range of an SU transceiver pair
*υ*	relative speed of SUs
$T_{PU}^{off,i}$	PU’s transmission period
$\tau_{on,i}$	PU’s arrival time
$\tau_{off,i}$	PU’s absence time
$P_{on,i}$	channel transition probability of ON to OFF state
$P_{off,i}$	channel transition probability of OFF to ON state
C	number of channels
$T_{s,i}$	period for spectrum sensing
$T_{sw,i}$	period for channel switching
$T_{i}$	channel access period
$r_{evt}$	event radius
$\bar{T_{r}}$	maximum route negotiation time
$R_{i}$	achievable spatio-temporal data rate on channel *i*
$\varpi_{i}$	spatio-temporal availability of channel *i*
$T_{su,i}$	packet transmission period
*θ*	scaling factor
$E_{th}$	SU’s node energy
$E_{th}^{\min}$	minimum allowed SU node energy
${clc}_{rreq}$	data channels in route request packet

It is important to note that this study only considers a network scenario of static PUs and mobile CRSN nodes. The scenario wherein mobile PUs and static SUs are considered [21] cannot be freely applied to the considered study. This is because, when the CRSN nodes are static in the presence of mobile PUs, the distance between the CRSN nodes (which is a prime function of accurate channel estimation when source-to-sink routing is considered) remains constant with time. This is quite different from the case where the CRSN nodes are mobile, which makes the channel estimation availability vary according to the distance of the next-hop selection, which also varies with time. Furthermore, PU protection, which is also a main concern of this work, was not considered in [21]. Table 1 presents a table of recurring symbols that have been used in developing and analyzing MROR.

## 4. MROR System and Network Model

In this section, the system and network model used for the design of MROR is explained. The section also presents the developed mobile routing channel availability model that captures the fluctuating nature of the spectrum opportunities in the mobility scenario. After developing the equations that characterize the spatio-temporal spectrum opportunities, the stay time for the OFF and ON states of the PU activity with respect to the mobile nodes is derived. Mobility-enhanced spectrum sensing considerations were also taken into consideration during the formulations.

### 4.1. Network Model

It is assumed that the nodes gain location awareness via an on-board GPS or by the implementation of a localization algorithm [22]. Unlike in [11,12], wherein the used mobility model was not mentioned, in this work, in order to reflect the concurrent influence of mobility and radio channels on the lifetime of a route, we utilized the smooth mobility model [23]. This model was chosen because it allows flexible, small, equal-length time steps (Δt) for smooth movement description. In addition to the fact that this model complies with the physical law of smooth motion, it has nice steady-state properties of a uniform nodal distribution and a stable moving speed, which fits the operation of deployed sensor networks.

The CR nodes are equipped with a half-duplex transceiver with CR capabilities. Based on a free-space path loss model, transmit power for both the PU and the SU is modeled to decay with distance. For each receiver-transmitter pair, the model considers both the signal-to-noise ratio (SNR) and receiver noise variance (RNV) based on the probabilistic wireless network simulator [24]. Thus, reception or detection is achievable only when the power limit from neighbors (*i.e.*, SUs) and primary users (PUs) is above a threshold, $\lambda_{su}$ and $\lambda_{pu}$, respectively.

The sensing mechanism for PU detection is the energy detection in a non-fading environment. When an SU performs spectrum sensing, the received signal, *S*, is [25]:

(1) $$S_{rec}^{su}=\left\{\begin{array}{cc} n(t), & \text{if}\;H_{0}\\ n\left(t\right)+S^{p}\left(t\right), & \text{if}\;H_{1} \end{array}\right.$$

where $H_{0}$ represents the PU hypothetical idle state, and the active state is represented by $H_{1}$. A zero-mean additive white Gaussian noise (AWGN), n(t), is added to the PU signal waveform, $S^{p}\left(t\right)$. Thus, the probability of detecting PU activity, $P_{d}$, and the probability for the occurrence of a false alarm, $P_{f}$, can be derived as [25]:

(2) $$P_{d}=\mathrm{Pr}\left\{Y>\lambda_{pu}\left|H_{1}\right.\right\}$$

(3) $$P_{f}=\mathrm{Pr}\left\{Y>\lambda_{pu}\left|H_{0}\right.\right\}$$

where $\lambda_{pu}$ represents the decision threshold for the decision statistic, *Y*, obtained from the energy detection algorithm. Hence, since the number of missed opportunities increases the false alarms, it implies that the probability of PU interference is increased by a low value of $P_{d}$, while a high value of $P_{d}$ will result in low spectrum utilization.

### 4.2. Mobility-Enhanced Channel Availability Modeling

The infrastructure-based primary network is assumed to be fixed with each cell comprising a base station and multiple receivers referred to as PUs. The PU network operates on a set of licensed channels C with similar temporal channel-usage statistics. Since the PUs are fixed, it is assumed that, both the (i) average density of PUs on each channel and (ii) temporal channel usage characteristics are known to the SUs. However, at any specific time, the SUs can only detect the availability of a channel via spectrum sensing. As highlighted, the proposed model considers, PU protection, channel estimation for mobile SU nodes and ensures the selection of stable routes. These entities are all jointly related as presented in the analytic formulations presented below.

#### 4.2.1. PU Protection

To ensure protection of PU communication, a primary transmitter keep-out radius is enforced by the regulatory body. Under the interference temperature limits (ITL), this distance is the minimum allowed distance between the transmitting PU and an SU under which an SU is not allowed to transmit.

(4) $$Z_{e,i}=\inf\left\{d\in Z\left|\sum_{\forall n}I\left(\delta_{s,i},d\right)\leq ITL\right.\right\}$$

where $\sum_{\forall n}I\left(\delta_{s,i,}d\right)$ represents the average interference generated by all of the *n* SUs on a primary receiver located at the edge of the coverage area of the primary transmitter on channel *i*; while $\delta_{s,i}$ represents the SUs density on the channel i. Accordingly, cumulative interference from SU to a primary receiver located at a distance $Z_{o}$ (*i.e*., located at the edge of the coverage of the primary transmitter) can be bounded as [26]:

(5) $$I_{i}^{U}\left(\delta_{s,i},Z_{e,i}\right)=\frac{2\pi P_{o}d_{o}^{\alpha }\delta_{s,i}}{\alpha -2}{\left(Z_{e,i}-Z_{o}\right)}^{2-\alpha }$$

where $P_{o}$ represents the SU’s transmission power, $d_{o}^{}$ is the reference distance, *α* represents the path-loss exponential, $Z_{o}$ the PU’s transmission radius, $Z_{e,i}$ the base station’s keep-out radius and $\delta_{s,i}$ is the average SU density on channel i. Thus, for Equation (5), we can define the primary transmitters’ keep-out radius necessary to achieve the interference constraint, $I_{i}^{U}\leq ITL$, on channel *i* as:

(6) $$Z_{e,i}^{*}\left(\delta_{s,i}\right)={\left[{\left(\frac{\alpha -2}{2\pi P_{o}d_{o}^{\alpha }\delta_{s,i}}\cdot ITL\right)}^{\frac{1}{2-\alpha }}\right]}^{+}+Z_{o}$$

From Equation (6), it can be observed that, for channel *i*, the keep-out radius increase with the PUs’ density. This basically means that, as more SUs access channel *i*, the keep-out radius has to be accordingly expanded to keep up with the ITL constraint. Since the formulation in Equation (6) is for a scenario wherein the PUs are stationary, it might not represent sufficient protection for the PU transmission in the mobile scenario, as the rate at which new nodes enter this region might increase depending on the speed of the SUs. Thus, this radius has to be adequately expanded to ensure PU protection in the mobile scenario. To achieve this, an addition protection layer referred to as the mobility-induced guard (mguard) distance, $\ell_{i}$, is introduced. When considered together with the ITL, we come up with a new mobility-induced protection (MIP) region, *ℑ*, for the primary transmitter. Thereby, if $P_{i}$ represents a set of transmitting PUs on channel *i*, the MIP region for a transmitting PU $\xi \in P_{i}$, located at $x_{i,\xi },y_{i,\xi }$, will represent the unit disc around ξ.

(7) $${ℑ}_{i,\xi }=\left\{\left(x,y\right)\in Z^{2}\left|∥\left(x_{i,\xi },y_{i,\xi }\right)-\left(x,y\right)∥\leq Z_{e,i}+\ell_{i}\right.\right\}$$

According to Equation (7), when an SU moves into any MIP region of an active PU, it can only initiate route formation if it is the source, and it can also compete in forwarding data packets if its instantaneous channel condition meets the session requirements of the active route.

In line with the analysis presented in [8] and taking into account the fact that a sequence of independent random variables, which are uniformly distributed, can be used to find a joint distribution, the average fraction of the summation of MIPs in the network for a channel will be:

(8) $${℘}_{i}\left(\delta_{s,i}\right)=1-e^{-\delta_{p,i}\pi {\left(Z_{e,i}\left(\delta_{s,i}\right)+\ell_{i}\right)}^{2}}$$

Hence, with a steady-state probability that, at any given time, the PU on channel *i* is not transmitting data, $\rho_{idle,i}=1-\rho_{busy,i}$, the average of the fraction areas where channel *i* is available can be approximated as:

(9) $$\Omega_{i}\approx \left(1-{℘}_{i}\right)+{℘}_{i}\rho_{idlef,i}=1-{℘}_{i}\rho_{busy,i}$$

#### 4.2.2. Channel Estimation

In other to derive the appropriate mobility channel availability model, we assume that (i) the statistic of the PUs’ traffic [27]; (ii) the time interval at which an SU moves in an MIP region [8]; and (iii) the time duration an SU remains within an MIP region all follow an exponential distribution [28,29]. According to [28], the duration at which an SU transceiver pair at a transmission range of ℜ moving at an average relative speed of *υ* remains within an MIP region can accurately be approximated as an exponential distribution attributed with intensity $\frac{\upsilon }{ℜ}.$ Prior to this, the time at which a mobile SU node first enters an active MIP region, $T_{in}$, can be approximated as [8]:

(10) $$T_{in,a}∼e^{\left(2\left(Z_{e,i}+\ell_{i}\right)\upsilon_{a}\delta_{p,i}\rho_{busy,i}\right)}$$

Based on the geographic forwarding scheme this work implements, the mobile route-aware channel availability model can take four possible states with respect to the SU’s mobility traffic pattern of the PU and the SUs’ location relative to MIP region and a local minima (LM) region. In geographical forwarding schemes, a node is said to have reached a local minima region when it cannot directly reach the sink while it is the closest to the sink with respect to all of its competing neighbors.

**Figure 1:**
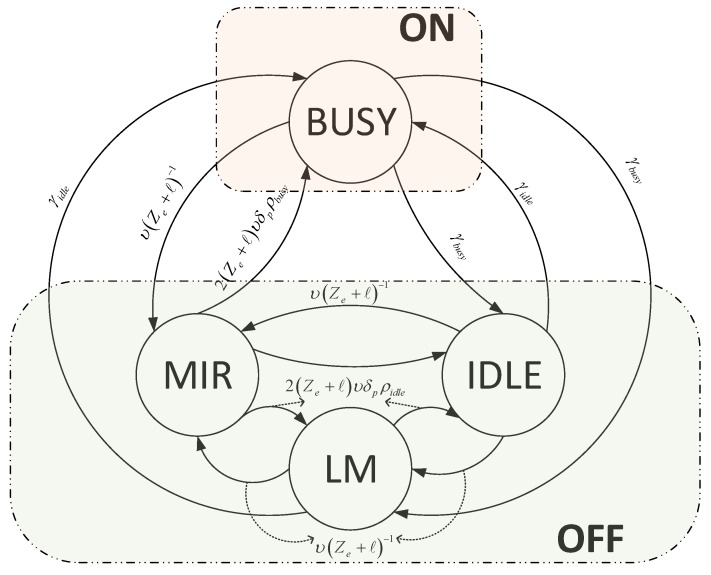
Mobile reliable opportunistic routing (MROR) channel availability model.

As shown in the Figure 1, the four states are the busy, idle, MIP and LM states. When a mobile SU moves into an LM or an MIP region of an active primary transmitter, the channel is assumed as not available (*i.e*., OFF state). However, when it is located outside any of the two regions, even if the primary transmitter is active, the channel is assumed to be available (*i.e*., ON state). Thus, by unifying the idle, LM and MIP states as the OFF state, the Markov chain can be simplified.

Accordingly, the distributions of the duration for the two states (*i.e*., ON and OFF) can be derived thusly: Considering a PU traffic resumption rate of $\gamma_{busy,i}$ on channel *i*; the stay time of the ON period follows an exponential distribution [28]:

(11) $$\tau_{on,i}∼e^{\left(\gamma_{busy,i}+\frac{\upsilon_{n}}{Z_{e,i}+\ell_{i}}\right)}$$

From Equation (11), based on the stationary distribution of the ON/OFF states that can be approximated from Equation (5) (*i.e*., $\rho_{on,i}=1-{℘}_{i}$ and $\rho_{off,i}={℘}_{i}$), using the detailed balanced equation, $\rho_{on,i}\gamma_{on,i}=\rho_{off,i}\gamma_{off,i}$, the state transition rate for OFF→ON, $\gamma_{off}$, results in:

(12) $$\gamma_{off,i}=\frac{{℘}_{i}\rho_{busy,i}}{1-{℘}_{i}\rho_{busy,i}}\left(\gamma_{busy,i}+\frac{\upsilon_{n}}{Z_{e,i}+\ell_{i}}\right)$$

From Equation (5), the stay time for the OFF state is derived:

(13) $$\tau_{off,i}∼e^{\left(\gamma_{off,i}\right)}$$

The sensing interval of an SU on any channel *i* depends on both the frequency of the PU activity on that channel and the distance it has traveled after the previous sensing operation [16]:

(14) $$t_{i}^{*}=\max\left\{T_{s,i},\min\left\{-\frac{\ln\left(1-\zeta \right)}{\gamma_{off}},\frac{\ell_{i}}{\upsilon }\right\}\right\}$$

where *ζ* is a predefined threshold, $\zeta \left(0<\zeta <1\right)$ that defines the cdf of the channel OFF state.

It is assumed that the licensed spectrum is utilized by PUs according to a probabilistic model. Since the PU arrival is independent, the ON and OFF states are exponentially distributed [30]. On arrival, the licensed user stays for $T_{PU}^{off,i}$ seconds. The ON and OFF states are respectively characterized by the arrival time, $\tau_{on,i}$ seconds, for the ON state and an absence time, $\tau_{off,i}$, for the OFF state. The transition of the ON to OFF state is with a probability, $P_{on,i}$, and it reverses its state (*i.e*., OFF to ON state) with a probability $P_{off,i}$.

(15) $$P_{on,i}=\frac{\tau_{on,i}}{\tau_{on,i}+\tau_{off,i}}$$

Hence, when $P_{on}$ is high, more PU activity on the licensed spectrum will be observed, which means fewer opportunities for SU transmission and *vice versa*. The OFF state probability will be:

(16) $$P_{off,i}=1-P_{on,i}$$

Once an SU commences data transmission on the data channel, it only releases the channel after it completes sending its packet. Suitable adoption of SU transmission power is used to control any interference that arises thereof [31].

For the sake of simplicity, it is assumed that a specific frequency band having C channels of the same bandwidth is chosen as the sensors’ operation area. Furthermore, all control signaling are via a dedicated common control channel (CCC), and the single transceiver is tuned to CCC by default and changes only during the data-forwarding phase.

Given that $T_{s,i}$ represents the time it takes to perform spectrum sensing and $T_{sw,i}$ represents the switching time to channel *i*, the average time an SU, *n*, has access to any channel, i∈C, will be:

(17) $$\omega_{n,i}=\left(1-\frac{\sum_{x=1}^{N_{n,s,i}}T_{s,i}-T_{sw,i}}{T_{i}}\right)$$

where $N_{n,s,i}$ represents the total number of times *n* performs spectrum sensing within the channel access time, $T_{i}.$

Finally, by multiplying the average time *n* has to utilize a channel *i* (*i.e*., Equation (17)) by the average fraction of areas where channel *i* is available (*i.e*., Equation (8)), we can arrive at the spatio-temporal availability of channel *i*, $\varpi_{i}=\Omega_{i}\omega_{i}.$

#### 4.2.3. Route Stability

In order to ensure the proper design that maintains a stable route, the optimal *mguard* distance $\ell_{i},$ that maximizes $\varpi_{i},$, has to be determined. Thus, since Equation (9) represents the average fraction of areas where channel *i* is available, it thus means that the fraction that is not covered can also be approximated as $\Omega_{i}\left(\ell_{i}\right)\approx e^{-f\left(\ell_{i}\right)}$, where $f\left(\ell_{i}\right)=\delta_{p,i}\gamma_{busy,i}\pi {\left(Z_{e,i}+\ell_{i}\right)}^{2}.$ With respect to the average OFF period, if we assume the overhead incurred on switching to be negligible, *i.e*., $T_{sw}\ll \gamma_{off}^{-1}$, then, $\omega_{i}$ can also be approximated as $\omega_{i}\approx 1-\frac{\upsilon T_{s,i}}{\ell_{i}}.$ Thus, the $\varpi_{i}$ can be written as:

(18) $$\varpi_{i}\left(\ell_{i}\right)\approx \Omega_{i}\left(\ell_{i}\right)\omega_{i}\left(\ell_{i}\right)\approx e^{-f\left(\ell_{i}\right)}\left(1-\frac{\upsilon T_{s,i}}{\ell_{i}}\right)$$

However, it can be shown that $\frac{\partial^{2}\Omega_{i}\left(\ell_{i}\right)}{\partial \ell_{i}^{2}}<0.$ Then, by setting the first-order derivative of $\Omega_{i}\left(\ell_{i}\right)$ to zero, we get:

(19) $$\frac{\partial^{2}\Omega_{i}\left(\ell_{i}\right)}{\partial \ell_{i}^{2}}=e^{-f\left(\ell_{i}\right)}\left(-2\gamma_{p,i}\rho_{busy,i}\pi \left(Z_{e,i}+\ell_{i}\right)\left(1-\frac{\upsilon T_{s,i}}{\ell_{i}}\right)+\frac{\upsilon T_{s,i}}{\ell {{}_{i}^{2}}_{}}\right)=0$$

In order to mathematically simplify the problem, $-2\gamma_{p,i}\rho_{busy,i}\pi \left(Z_{e,i}+\ell_{i}\right)$ in Equation (19) approximates to zero to give us the quadratic equation:

(20) $$\left(Z_{e,i}-\upsilon T_{s,i}\right)\ell_{i}^{2}-Z_{e,i}T_{s,i}\ell_{i}-\frac{\upsilon T_{s,i}}{2\gamma_{p,i}\rho_{busy,i}\pi }=0$$

After solving Equation (20), the optimal *mguard* distance, $\ell_{i}$, that maximizes $\varpi_{i}$ can be given as:

(21) $$\ell_{i}^{*}=\frac{T_{s,i}\upsilon Z_{e,i}+\sqrt{{\left(T_{s,i}\upsilon Z_{e,i}\right)}^{2}+{\frac{2T_{s,i}\upsilon \left(Z_{e,i}-T_{s,i}\upsilon Z_{e,i}\right)}{\pi \delta_{p,i}\rho_{busy,i}}}^{}}}{2\left(Z_{e,i}-T_{s,i}\upsilon \right)}$$

### 4.3. Channel Pool Update

**Figure 2:**
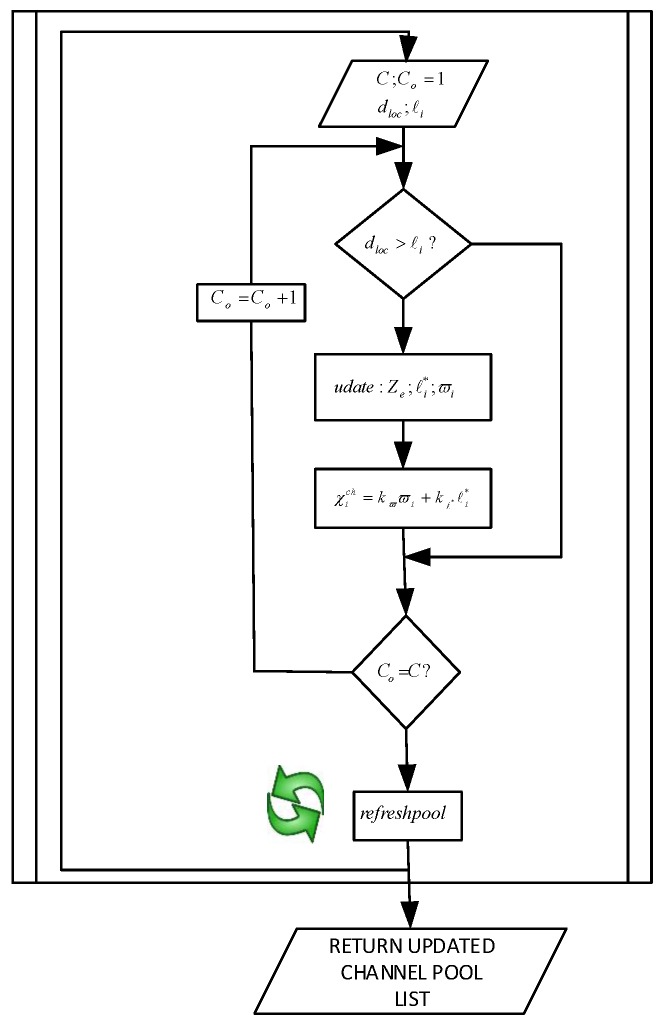
MROR channel pool update algorithm.

Every node keeps a pool of channels Cchannels, which is continuously updated based on the $\varpi_{i}$ of each channel and the proximity of the mobile node with respect to the relative value of $\ell_{i}^{*}.$ As represented in Equation (22), both metrics are then formulated into a channel selection cost function, $\chi_{i}^{ch}$; where $k_{\varpi }$ and $k_{\ell^{*}}$ are normalizing constants. Thus, the channel having the highest value of $R_{i}^{ch}$ will be on top of the pool. Figure 2 illustrates the full operation of the channel update algorithm.

(22) $$\chi_{i}^{ch}=k_{\varpi }\cdot \varpi_{i}+k_{\ell^{*}}\cdot \ell_{i}^{*}$$

## 5. MROR: Mobile Reliable Opportunistic Routing for CRSN

This section presents the details of MROR operational blocks. MROR is made up of a route request module, a route selection module, a VCG formation module, a VMH zoning strategy, a VCG initiative determination forwarding (VIF) and route management modules.

### 5.1. Channel Selection and Route Request Initiation

We assume that an event area of radius $r_{evt}$ meters is to be monitored by the mobile sensors. A typical event can be post-disaster rescue missions or military actions, where the SUs corresponding to mobile robots are strategically deployed to move into difficult and wide terrain to monitor and send their sensory readings to a remote sink. The sensors transmit their data in the form of pictures, low-resolution videos, temperature or a selective combination of these entities to a remote sink.

When potential source nodes hit a target area, they contend in initiating a route request by broadcasting a route request (RREQ) packet. Once a node successfully gets through its route request, other nodes will desist route request broadcast and only participate in the implementation of the route request activity. The RREQ packet encapsulates the source’s address, the sink’s address along with the RREQ tag. After the broadcast, the source node initiates a timer, $\bar{T_{r}}$. This timer carries the maximum route negotiation time. Within this time, the source node expects to receive a route reply message. If the source node times out without receiving any reply, another RREQ is sent. Depending on the kind of media being transmitted, a quality of service requirement is stipulated by the source node to successfully send the traffic to the sink. This value is also embedded in the route request payload. For this study, the considered QoS metric is the minimum data rate essential to service available packets. This information is captured by the application layer, which is the layer that is in direct contact with the activated sensor. Although the function $\chi_{i}^{ch}$ can be used to identify an appropriate channel for data transmission, it is pertinent to note that $\chi_{i}^{ch}$ does not consider the bandwidth and the spectrum sensing error, which are equally important metrics for ensuring QoS. Thus, to be sure a chosen channel can support the traffic at hand, confirming its data rate is essential. While considering mobility, the required data rate will be defined as the achievable spatio-temporal data rate, $R_{i}$, on any channel based on its spatio-temporal availability, $\varpi_{i}$, which depends on both the speed and the transmission range of the node $\frac{\upsilon }{ℜ}$. This defines the sum of the rate, $R_{s,i}\left(\varpi \right)$, when a channel is detected idle alongside the possibility of PU appearance during the period of packet transmission, $T_{su,i}$, with the achieved rate, $R_{f,i}\left(\varpi \right)$, because of false channel availability. Note that interference among SUs, $I_{su,i}$, is considered in both cases. Hence, the attainable data rate when the PU is inactive and there is no false alarm can be represented as:

$$b_{s,i}\left(\varpi \right)=\beta log_{2}\left(1+\frac{S_{rec}^{su}}{n_{i}+I_{su,i}}\right)$$

(23) $$R_{s,i}\left(\varpi \right)=\left(P_{off,i}-P_{f,i}\right)\frac{T_{su,i}-\bar{T_{s,i}}-\bar{T_{r}}}{T_{su,i}}b_{s,i}\left(\varpi \right)$$

However, a PU can arrive at any time during the time $T_{su,i}$, which subsequently results to interference that converges to $\left(1-P_{off,i}\right)T_{su,i}$ with a probability $1-e^{-\theta \frac{T_{su,i}}{\tau_{on}\varpi_{i}}}$, where *θ* is a scaling factor. On the other hand, the achievable data rate, $R_{s,i}\left(\varpi \right)$, in the case where, due to spectrum sensing error, the PU is not detected, although it is active, is:

$$b_{f,i}\left(\varpi \right)=\beta \log_{2}\left(1+\frac{S_{rec}^{su}}{n\left(\varpi \right)+S^{p}\left(\varpi \right)+I_{su,i}}\right)$$

(24) $$R_{f,i}\left(\varpi \right)=\left(P_{on,i}-P_{d,i}\right)\frac{T_{su,i}-\bar{T_{s,i}}-\bar{T_{r}}}{T_{su,i}}b_{f,i}\left(\varpi \right)$$

Thus, the attainable spatio-temporal data rate, $R_{i}$, on a channel is:

(25) $$R_{i}\left(\varpi \right)=e^{-\theta \frac{T_{su,i}}{\tau_{on,i}\varpi_{i}}R_{s,i}\left(\varpi \right)}+\left(1-e^{-\theta \frac{T_{su,i}}{\tau_{on,i}\varpi_{i}}}\right)R_{f,i}\left(\varpi \right)$$

where $e^{-\theta_{1}}\frac{T_{su,i}C}{\tau_{on,i}\varpi_{i}}$ is the probability that the PU keeps transmitting on the channel throughout the entire packet transmission time $T_{su,i}$.

**Figure 3:**
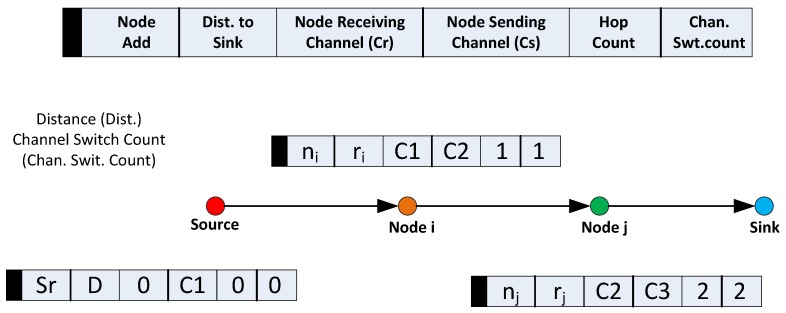
Route request (RREQ) operation with payload.

Depending on the kind of sensor activated on the source node, the application layer encapsulates the minimum required data rate into the route request payload. Alongside, it encapsulates its distance from the sink (POS) and its chosen data channel. It is important to note that, in the case of the source node, both the node’s receiving channel field, Cr, and sending channel field, Cs, will be the same. This is illustrated in Figure 3. Contrarily, as will be discussed in the next session, the Cr and Cs fields of all other nodes may vary. Finally, the source sets the channel switch count and hop count to zero. Afterwards, it will broadcast the packet via CCC.

### 5.2. Route Request

When a node, *i*, receives an RREQ from another node, which is here referred to as the “requesting node”, in order to be sure that the same packet has not been previously received, node *i* checks the tag on the packet. Node *i* drops the packet if has been received previously. Otherwise, it proceeds to implement the eligibility determination algorithm. If its eligibility (*El*) determination operation yields a “1”, node *i*, which now becomes a requesting node, will re-broadcast the RREQ packet. If, on the other hand, (*El*) returned a zero, the packet is dropped. (*El*) is fully a binary operation, which is determined as below:

(26) $$El=\left\{\begin{array}{c} 1,\left\{\begin{array}{c} d_{sk}<d_{sk}^{req}\\ E_{th}\geq E_{th}^{min}\\ clc={clc}_{rreq}\\ R\left(\varpi \right)\geq R_{\varpi_{t}} \end{array}\right.\\ \text{else}\;=\;0 \end{array}\right.$$

Node *i*, has to meet four conditions before its *El* operation returns a one. These conditions include the following. Its POS has to be smaller in value than that of the requesting node, *i.e*., $d_{sk}$ has to be smaller than $d_{sk}^{req}$. This condition ensures the packet keeps a forward flow to the sink. The condition $E_{th}\geq E_{th}^{\min}$ is used to check if the energy of node *i*, $E_{th}$, is above the minimum value, $E_{th}^{\min}$. Node *i* uses the third condition, $clc={clc}_{rreq}$, to check if the offered data channel in the ${clc}_{rreq}$ field is available. The last condition, $R\left(\varpi \right)\geq R_{\varpi_{t}}$, helps node *i* to confirm if the spatio-temporal characteristic of the chosen channel in ${clc}_{rreq}$ supports the needed traffic. At this point, if the RREQ channels passes node *i*’s local rate test, it is retained in ${clc}_{rreq}$ as its chosen data channel. Thus, the value for the channel switch count will not be altered. On the other hand, if node *i* has another channel that exhibit better performance, it is replaced, and the channel switch count is incremented by “1”. This thus means that different channels will be used by node *i* for its upstream and downstream communication. Node *i* will then cache the channel(s) and position to the sink (POS) of the requesting node. If the path was finally selected as the best path by the sink, this requesting node will be known as node *i*’s upstream LMR node. Before the node *i* re-broadcasts the RREQ packet, it performs some bookkeeping tasks as follows: it replaces the requesting node’s POS with its POS and increments the hop count. If, on the other hand, none or any of the conditions were met by node *i*, it immediately drops the RREQ packet. This adopted strategy helps keep the RREQ packet short, as against the method used in [9], wherein the size of RREQ packet grows together with the hop count. This route request is continued until all possible disjoint routes are discovered.

### 5.3. Route Selection

Once the first RREQ packet arrives at the sink, the sink sets a timer. The sink keeps accepting new route requests until the timer times out; after which, any arriving route request packets are declared out-of-date and subsequently discarded. It is inferred that the late arrival of such requests indicates the inferior quality of the corresponding paths. Two metrics are extremely considered in the final route selection; the channel switching and hop counts. Both are worked into the route cost-function $\chi_{rut}$. This function favors routes with low channel switching by declaring half the weight of the hop count metric for the channel switch metric.

(27) $$\chi_{rut}=k_{h}\cdot W_{h}+\frac{k_{s}}{2}\cdot W_{s}$$

where $k_{h}$ is the assigned weight for the hop count and $k_{s}$ is the assigned weight for hop count channel switch count, along the path. $W_{h}$ and $W_{s}$ are, respectively, the path’s hop count and the channel switch count. The path having the least cost is the final one, and all nodes on the path are subsequently referred to as the path’s local minimum resolution (LMR) nodes.

### 5.4. VCG Formation

**Figure 4:**
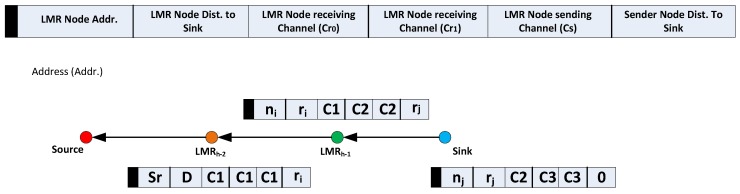
Virtual contention group (VCG) formation operation with payload. LMR, local minimum resolution.

On the conclusion of route selection, from the sink, the nodes at each hop along the chosen path are tagged as $LMR_{\left(h-1\right)}$, $LMR_{\left(h-2\right)}$, …, $LMR_{0}$. As illustrated in Figure 4, the sink will then generate the VCG formation packet and broadcast it via CCC to the source node. The actions of other nodes that receive the packet while not being next hop nodes include reading the packet perform the VCG grouping check, as explained in the next paragraph. Meanwhile, the only node having the right of rebroadcasting the VCG packet has its address encapsulated in the packet.

Before the sink node broadcasts the VCG formation packet, it includes the following information in the packet: the address of the next upstream LMR node, $n_{j}$, $\left(LMR_{\left(h-1\right)}\right)$ and $n_{j}$’s POS, $r_{j}$, on the VCG payload. It will be recalled that the same information was earlier acquired during the route request phase. In addition, the sending nodes’ POS (for the sink, this value will be zero) is also added on the VCG payload. As discussed in Section 5.2, if $n_{j}$ have separate upstream and downstream channels, the Cr0 and Cr1 fields are correspondingly replaced with Cr and Cs, which were earlier acquired during the RREQ phase; while the Cs field retains the value of Cs. This configuration significantly expands the possible reception area and, in effect, still reduces the final hop count. Based on geographical routing schemes that utilize distance and the signal-to-noise reception ratio (SNR) as forwarding metrics, nodes in the transitional region have been observed to usually show the maximum (joint distance and SNR) forwarding values when lossy links are considered [32]. The VCG formation payload, as illustrated in Figure 4, also carries the required data rate. The functions of other fields of the VCG payload are that the VCG packet identifier differentiates control packets and also helps notify all non-next-hop nodes between two consecutive LMR nodes to perform the VCG formation operation. The address of the next upstream LMR node identifies the node expected to rebroadcast the VCG formation packet. Furthermore, the information of a sender node’s POS and the LMR node’s POS is utilized by all nodes between two consecutive LMR nodes to determine their instantaneous position.

When the nodes determine their locations, if the nodes’ locations fulfill $d_{sk}^{lmr}\geq d_{sk}\geq d_{sk}^{snd}$, then they will proceed with the following set of operations to determine the VCG eligibility operation; where $d_{sk}^{lmr}$ is the next upstream LMR node’s POS, $d_{sk}^{snd}$ represents the sending node’s POS and $d_{sk}$ represents the receiving node’s POS.

**Figure 5:**
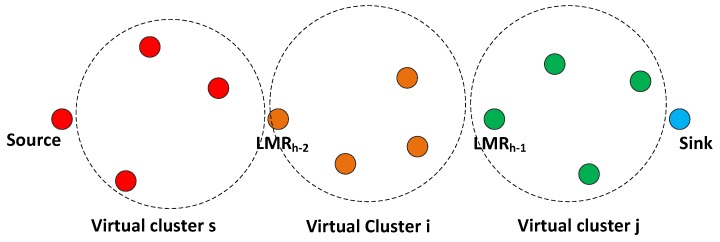
VCG organization after the VCG formation stage. VCG, virtual contention group; PU, primary user.

As implemented for the route formation stage (Section 5.1), the VCG eligibility operation is performed by any node that receives a VCG packet. The difference in this case lies in the first condition, $d_{sk}^{lmr}\geq d_{sk}\geq d_{sk}^{snd}$. This condition ensures that only eligible nodes located between the broadcasting node and the next downstream LMR node return a positive value. This condition is considered alongside the three criteria defined in Equation (26). As illustrated in Figure 5, once a node returns a positive value for its VCG eligibility operation, it becomes a member of the virtual cluster of the LMR node’s address indicated in the VCG payload. After associating with a cluster, the nodes will read the channel assignments and subsequently cache them in their routing tables. They then switch their transceiver to channel Cr0 to begin the data forwarding phase.

(28) $${El}_{vcg}=\left\{\begin{array}{c} 1,\left\{\begin{array}{c} d_{sk}^{lmr}\geq d_{sk}\geq d_{sk}^{snd}\\ E_{th}\geq E_{th}^{min}\\ clc={clc}_{rreq}\\ R\left(\varpi \right)\geq R_{\varpi_{t}} \end{array}\right.\\ \text{else}\;=\;0 \end{array}\right.$$

The next LMR node to receive the VCG packet inserts the required VCG information and broadcast the packet through CCC. Afterwards, its transceiver is switched to its Cr. All subsequent VCGs are formed in this manner, until the VCG packet is received by the source. A still-alive packet is periodically geocast by an LMR node to show the active state of a VCG region.

### 5.5. VMH Zoning System

In order to ensure the stability of the built routes, MROR introduces the concept of the virtual mobile handover (VMH) zoning system. VMH defines an area in the just created virtual clusters beyond which an LMR node has to hand over its role to the most deserving node in the virtual cluster. Once the LMR nodes have been identified, the VHM initiator function is automatically activated. This function helps the LMR node when it is appropriate for it to initiate the handover process to the next most deserving node in its virtual cluster. The VHM initiator function is formulated as:

(29) $$\frac{\upsilon_{n_{i}}}{0.6\left(d_{sk}^{lmr_{h-a}}-d_{sk}^{lmr_{h-a+1}}\right)-\left(d_{sk}^{n_{i}}-d_{sk}^{lmr_{h-a+1}}\right)}\leq t_{ho}$$

where $t_{ho}$ is the expected time it takes for a successful handover operation. Due to the speed of the node, this function was designed to be primarily triggered when an LMR node has covered 60% of its VC. This value was chosen because, since the protocol was designed to always select routes with the lowest channel switch, at 60% of the previous VC, the system gives more chances to the handing over node to still participate in forwarding packets before it enters into the next VMH zone. Thus, $\upsilon_{n_{i}}$ represents the speed of the node *i*, $d_{sk}^{lmr_{h-a}}$ is the distance-to-sink of the handing over node at the time the VCG formation ended. Likewise, $d_{sk}^{lmr_{h-a+1}}$ is the distance-to-sink of the preceding LMR node at the time the VCG formation ended, and $d_{sk}^{n_{i}}$ is the present distance-to-sink of node i. *a* is used to represent the VC id in which the handover is being initiated.

On the other hand,

(30) $$t_{ho}=\frac{2t_{s,hp}+t_{w,rp}+t_{t/o}}{p_{s}^{2}}$$

where $t_{s,hp}$ is the expected time it takes the handing over LMR node to successfully announce the handover bidding process. This value is multiplied by two, because a handover exercise is only successful after the LMR node receives a broadcast reply from an eligible node, which marks the end of the handover process. The expected time the node will wait for a reply is represented by $t_{w,rp}$, and $t_{t/o}$ is the expected time the node’s timer runs out (*i.e*., deciding that a neighbor does not exist). This subsequently triggers the route maintenance module, as discussed in Section 5.8.

**Figure 6:**
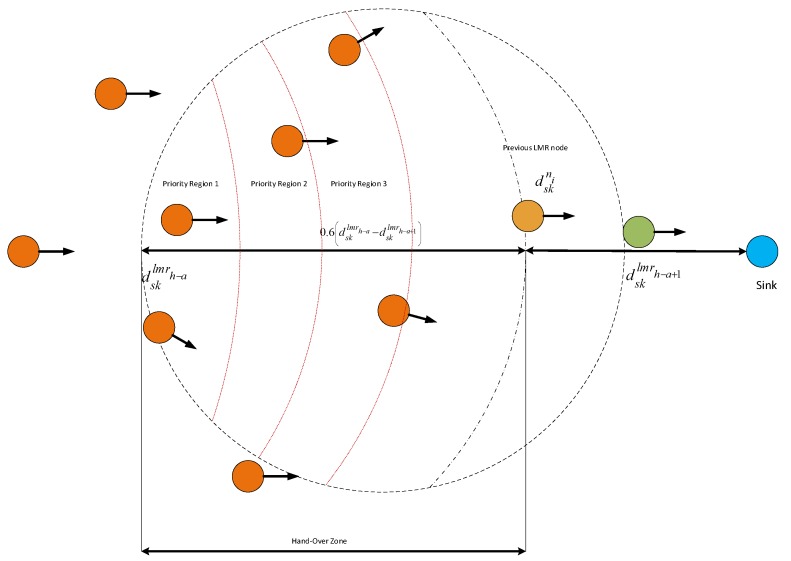
MROR virtual mobile handover zoning system.

As illustrated in Figure 6, once an LMR node successfully broadcasts its handover request packet (hrp), the region between $d_{sk}^{n_{i}}$ and $d_{sk}^{lmr_{h-a}}$, which is referred to as the handover region, is partitioned into *G* priority regions. Every priority region ${pr}_{i}$ is made to match a back-off window size $CW_{i}$. Thus, based on a node’s location in the handover region, it backs-off for $\sum_{j=1}^{i-1}CW_{j}+cw_{i}$, where $cw_{i}$ is randomly selected, such that $cw_{i}\in \left[0,CW_{\max}\right]$ where $CW_{\max}CW_{i}-CW_{i-1},\forall_{i}.$ Hence, based on how close a node is to $d_{sk}^{lmr_{h-a}}$, this backoff scheme prioritizes them. When a node receives an hrp packet, it backs-off for $\sum_{j=1}^{i-1}CW_{j}+cw_{i}$, as corresponds to its priority area. In this way, a node in any priority region will wait in its priority slot for an average of $CW_{\max}/2\;$ in addition to the waiting period of all of the previous priority slots. During this period, if it overhears a handover reply from another node, it assumes the LMR position as occupied and refrains from sending. At this stage, the handover process is declared completed.

In arriving at a value for $t_{ho}$, a successful broadcast of the handover request packet (hrp), $p_{s}$, depends on two probabilities: (i) a successful carrier sense, $p_{cs}$; and (ii) no collision occurred during packet transmission, $p_{coll}$:

(31) $$p_{cs}={\left(1-(1-p_{cf})\right)}^{k+1}$$

where *k* is the number of re-sensing allowed for a specific transmission and $p_{cf}$, represented below, is the probability of sensing the channel as free:

(32) $$p_{cf}=e^{-\lambda {}_{net}\left(t_{cs}+t_{prg}\right)}$$

$t_{cs}$ is the period for carrier sensing. A collision can only occur when a second node tries transmitting during $t_{cs}$. Thus, the probability of experiencing no collision can be computed as:

(33) $$p_{coll}=e^{-\lambda {}_{net}t_{cs}}$$

$\lambda {}_{net}$ represents the overall traffic generated by all nodes inside the VC (which is a summation of the original traffic, *λ*, and the traffic generated due to retransmissions).

(34) $$\lambda_{net}=\lambda \frac{p_{cs}}{p_{prg}}\left(1-{\left(1-p_{prg}\right)}^{L+1}\right)$$

*L* is the number of allowed retransmission, while $p_{prg}$ represents the probability that the node sending the hrp packet can successfully access the channel. This can be computed by solving $p_{cs}p_{coll}.$

**Figure 7:**
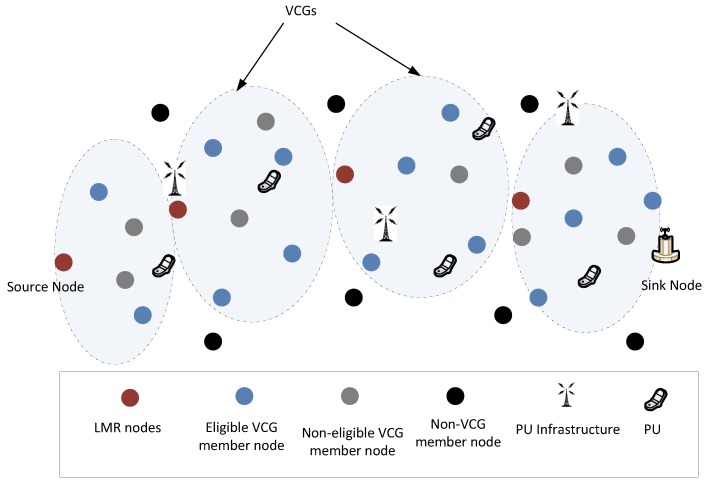
Full network illustration for a mobile reliable opportunistic routing (MROR)-based cognitive radio sensor network (CRSN).

Thus, if $t_{prg}$ is the propagation for the hrp packet to travel in air from the LMR node to all eligible nodes in the VC, then $t_{s,hp}=t_{prg}{\left(p_{cs}p_{coll}\right)}^{2}$, $t_{s,hp}=t_{prg}\left(1-{\left(p_{cs}p_{coll}\right)}^{2}\right)$ and $t_{w,rp}=t_{prg}\left\{\sum_{i=1}^{G}\left[\left(\sum_{k=1}^{i-1}CW_{k}\right)+\frac{CW_{\max}}{2}\right]{\left(p_{cs}p_{coll}\right)}^{2}\right\}$. Figure 7 illustrates the full network model for MROR.

### 5.6. VCG-Based Data Forwarding Initiative Determination

The data forwarding in MROR is based on VCG receiver-based contention. After all initialization processes, all nodes switch to the data channel (as determined from the VCG formation phase) in preparation for the forwarding phase. A node that has a data packet encapsulates its location information and its VCG LMR node’s tag on the data packet and then broadcasts the packet afterwards. The data initiative forwarding determination (DIFD) operation is then performed by all eligible VCG nodes that received this data packet successfully. The DIFD binary operation is performed as follows:

(35) $$I_{DIFD}=\left\{\begin{array}{c} 1,\left\{\begin{array}{c} d_{sk}\leq d_{sk}^{fd}\\ B>B_{th}\\ \xi_{pkt}\geq \xi_{th}\\ R\left(t\right)\geq R_{t} \end{array}\right.\\ \text{else}\;=\;0 \end{array}\right.$$

A node’s initiative is regarded as successful only if the $I_{DIFD}$ operation returns a one. Such a situation can only arise if the receiving node’s POS, $d_{sk}$, is less than $d_{sk}^{fd}$, which is the forwarding node’s POS. This condition ensures two important issues: (i) that the packet maintains a progressive movement to the sink; and (ii) to eliminate the possibility of the occurrence of an infinite routing loop. The condition $B>B_{th}$ is the local congestion control parameter. It prevents packet drops (*i.e*., buffer overflow) by ensuring that relay node’s buffer occupancy level, *B*, does not exceed the acceptable threshold, $B_{th}$. This metric also helps ensure that network load is fairly distributed among potential eligible relay nodes. An unbalanced network load distribution can occur when hot spots are created. This is when a node becomes a favorite relay node, due to its position in the network. Thus, the generated traffic will favorably be channeled to this node, which usually results in a buffer overflow in relay nodes. This, consequently, results in adverse end-to-end network performance. Thus, with this condition, $B>B_{th}$ ensures the network load distribution by giving an opportunity to equally eligible nodes to relay packet. The third condition is the link quality indicator, $\xi_{pkt}\geq \xi_{th}$; the third condition is used to always ensure the availability of a reliable link. It ensures that any relay node must have received the packet at a signal-to-noise ratio of $\xi_{pkt}$, which is above the set threshold, $\xi_{th}$. Otherwise, $I_{DIFD}$ returns a null. If a positive result is returned after completing the $I_{DIFD}$ operation, the successful node will delay sending its generated acknowledgment (ACK) packet according to a scheme referred to as the receiver contention prioritization scheme.

### 5.7. Receiver Contention Prioritization

In the design of the receiver contention prioritization scheme, more priority is granted nodes that make better advancement towards the sink, to transmit the packet. With respect to the location information, the contention region is divided into Q priority regions, *i.e.*, $A_{i},$ where i=1,2,...,Q. Nodes that return a positive value for the $I_{DIFD}$ operation determine their priority regions based on the location information stored in the header of the data packet. Afterwards, according to their respective priority regions, each node delays sending its ACKpacket.

**Figure 8:**
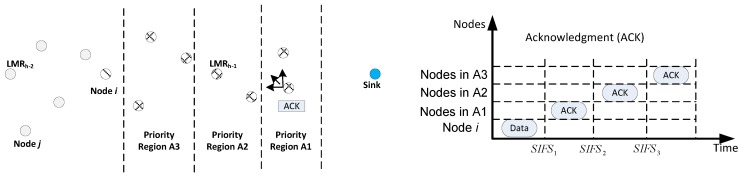
Receiver contention prioritization and backoff scheme.

A delay window size of short inter-frame space (SIFS), $SIFS_{i}$, is tagged to each priority region, $A_{i}$. Accordingly, all potential next hop nodes will delay for $\sum_{j=1}^{i-1}SIFS_{j}+sifs_{i}$; where $sisf_{i}$ is randomly chosen, such that $sifs_{i}\in \left[0,EIFS\right]$, where the extended inter-frame space $\left(EIFS\right)=SIFS_{i}-SIFS_{i-1},\forall i$. This configuration implies that nodes that will compete to relay the packet will be only those that reside in the same priority region. Once a contesting node successfully sends its ACK packet, all nodes undergoing delay will immediately drop the data packet when they overhear the ACK packet. The ACK is used to notify all of the delaying nodes about the successful selection of a next-hop node. Figure 8 is used to present a case for three priority regions.

When node *i* intends to forward a data packet, it simply broadcasts the packet through its data channel; nodes that returned a positive value for $I_{DIFD}$ initialize their SIFS according to their priority region (Figure 6). Among all of the regions, nodes within the region $A_{1}$ will first contend in broadcasting the ACK packet after $SIFS_{1}$ seconds. When a node is successful, others refrain from broadcasting ACK and subsequently drop the packet. In a case where no ACK is received by the delaying nodes after $SIFS_{2}$ seconds, nodes in $A_{2}$ commence to compete in sending their ACK, and so on. Note that organizing the nodes in VCGs helps to prevent the event of two nodes sending an ACK packet without hearing each other. However, the collision of ACK packets from the same region may occur. This case results in ACK being sent by a lower-priority node. MROR does not resolve this problem because the cost of resolving such outweighs the gains. Moreover, such an occurrence is very low, because the scheme segregates nodes into contention regions.

On the other hand, it is also possible that node *i* did not receive an ACK. This can occur as a result of the following: (i) due to mobility, the eligible node falls into a local minimum area; or (ii) due to the failure of the node;s neighbors. In such a case, node *i* makes *k* attempts, after which it concludes the occurrence of a local minimum situation. It afterwards switches to the route maintenance module, which helps in resolving the issue.

### 5.8. Route Maintenance

In order to detect the local minimum problem, node *i* compares the distance-to-sink of its immediate downstream node, node *j* with the POS of its LMR node. If the POS of node *j* is less than the POS of its LMR node, the packet is sent back to node *j* by piggybacking a local-minimum-alert message. Once the packet is successfully received by either node *j* or the LMR node, node *i* afterwards switches its status to a non-eligible VCG member node. The node that received the local minimum alert message will become responsible for seeking a different path for the packet via VCG-based data forwarding initiative determination, as discussed in Section 5.4, and receiver contention prioritization, discussed in Section 5.6.

Another possibility that can occur is the failure of an LMR node. The failure of an LMR node can be detected when the VCG members did not receive a still-alive packet for *z* consecutive times. At this instance, the VCG member node that is closest to the previous LMR node becomes the next LMR node. This is achieved by making all of the VCG members initiate a random timer, NLMR, having a value between $CW_{\min}$ and $\left(CW_{\min}+{\left(\frac{CW_{\min}}{2}\right)}^{a}\right)$. The node whose timer expires first announces itself as the LMR node, thus reflecting its proximity to the position of the previous LMR node. It should be noted that the scheme is designed such that LMR nodes only take part in packet forwarding if they happen to be the best nodes at that instance fulfilling the forwarding criteria; as a result, failure of an LMR node is usually a rare occurrence.

**Figure 9:**
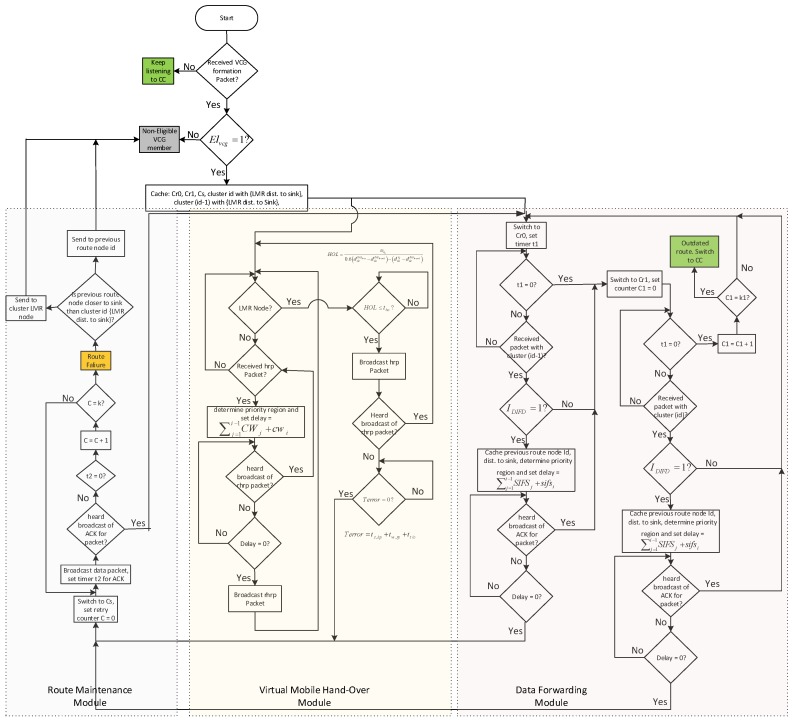
Flow chart for the MROR node forwarding algorithm. CCC, control channel; SIFS, short inter-frame space; DIFD, data initiative forwarding determination.

In addition, although for the worst case scenario, nodes have to switch between receiving and sending channels, it has been noted that not synchronizing this process results in more efficiency for the system. This is because the distributed nature of the scheme always ensures the availability of a potential forwarding node without jeopardizing the downstream flow in any way. Moreover, as discussed in Section 5.3, during the route selection phase, the scheme aims at minimizing the occurrence of multiple channel switching. Figure 9 shows the full MROR algorithm flow.

## 6. Performance Evaluation

**Figure 10:**
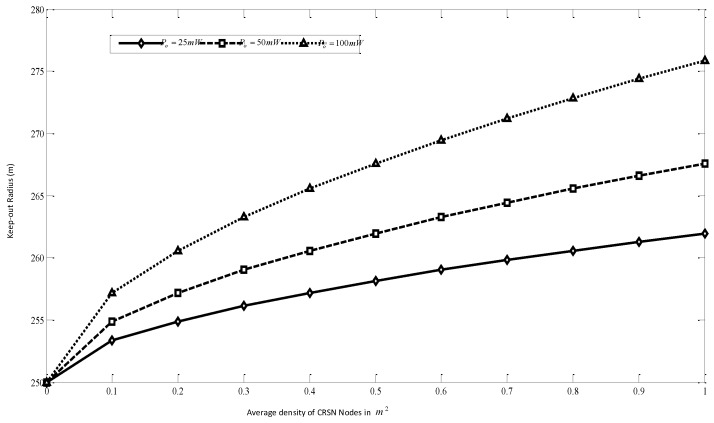
Effect of keep-out radius in routing for cognitive radio sensor networks (CRSNs).

From the system model, certain observations were made. From Equation (6), as can be viewed from the Figure 10, in order to ensure both PU protection and the route stability (for routes outside the PU coverage), the density of the keep-out radius has to be increased as the number SU users increases. Since the mobility of nodes increases the chance of an increase in SU density in time, it is proposed that routes that are formed outside PU coverage should be aware of the mobility-induced guard (mguard) distance in addition to the keep-out distance, as formulated in Equation (21). The simulation parameters considered for this case include: $Z_{o}$ = 250 m, ITL = 0.1 mW, *α* = 3.8 and $\delta_{p}$ = 1/km${}^{2}$.

**Figure 11:**
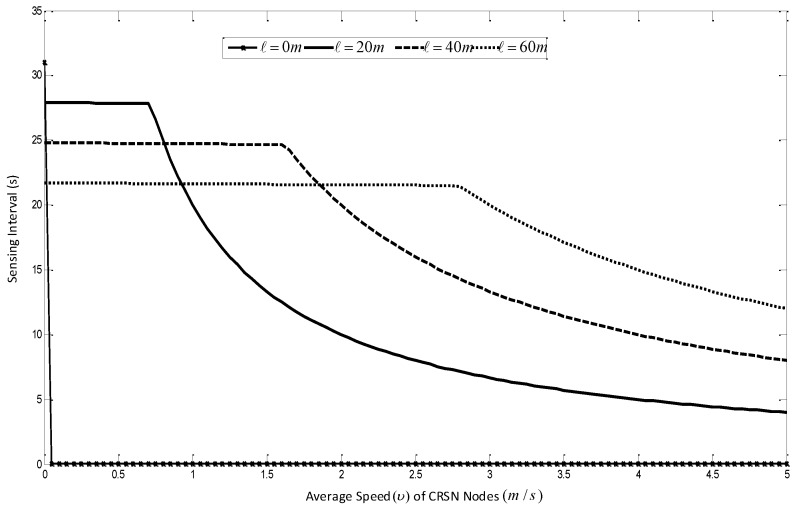
Effect of speed on spectrum sensing intervals in a cognitive radio sensor network (CRSN).

**Figure 12:**
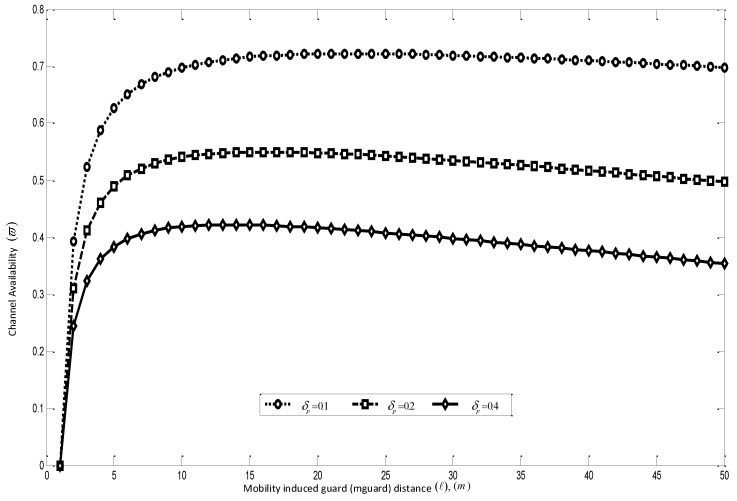
Mobility-induced spectrum availability in a cognitive radio sensor network (CRSN).

Secondly, as derived from the Equation (14) it is clear from Figure 11 that at a higher speed, the sensing interval is more dependent on the speed of the mobile nodes than it is on the statistics of the PU traffic. This is a major point that has not been considered in other mobile *ad hoc* protocols. When considered, this property has the potential of significantly increasing the route throughput and also to save energy, as the number of times the CRSN node has to perform spectrum sensing can be reduced by the nature of the routes formed. This fact is further corroborated in Equation (18), as can be seen in Figure 12, because depending on the number of PU users in the network, the channel availability can be said to become stable only at certain values of $\ell_{i}$. For the simulation in Equations (14) and (18), the following parameters were set thusly; $Z_{o}$ = 250 m, *υ* = 5 m/s, $\delta_{s}$ = 0.1, $\delta_{p}$ = 1/km${}^{2}$ and $P_{on}$ = 0.6.

The communication complexity in MROR is evaluated and compared to ROR and CRP in the MATLAB-based routing modeling application simulation environment (Rmase) [33,34] of the probabilistic wireless network simulator (Prowler) [24,35]. A topology of 40–200 mobile cognitive radio sensor nodes, which are uniformly deployed in a 100 × 100 m${}^{2}$ sensor network, is considered. The sensors are distributed such that they cut across PU coverage. The sink is considered to be static and located with coordinates (120, 120), away from the coverage of the PU network. For each considered case of simulation, an event at coordinates (20, 20) having an event radius of 20 m${}^{2}$ is reported to the sink node. From the seven channels available, each node makes a selection, as discussed earlier during the RREQ phase. All of the channels are modeled to possess the same bandwidth with overlapping with adjacent channels. This ensures that, during transmission, interference does occur between packets on different channels. In relevant scenarios, results were taken for values of $P_{on}$ between 0.2 and 0.6. The duration for each simulation was set for 100 s. The final results presented are average values of 10 trials along with 80% confidence intervals.

The outlined performance metrics were investigated:

- Throughput: This metric is used to measure the time performance of the considered application. The throughput is computed as the number of bits-per-second received at the sink. Since certain protocols allow multiple copies to reach the sink, thus unique packets out of the total packets the sink receives are considered in computing the throughput.
- Goodput: Goodput is a measure of the reliability of communication in the considered network. It is computed as the ratio of total unique packets the sink receives by the number of packets sent from the source nodes of each stream.
- Energy efficiency: Energy efficiency is computed as the ratio of the number of packets the sink receives by the total energy consumed by the protocol in the network.
- Route Stability Ratio: Route stability ratio is the ratio of unsuccessful routes between each node in the network to the number of all possible routes.

**Figure 13:**
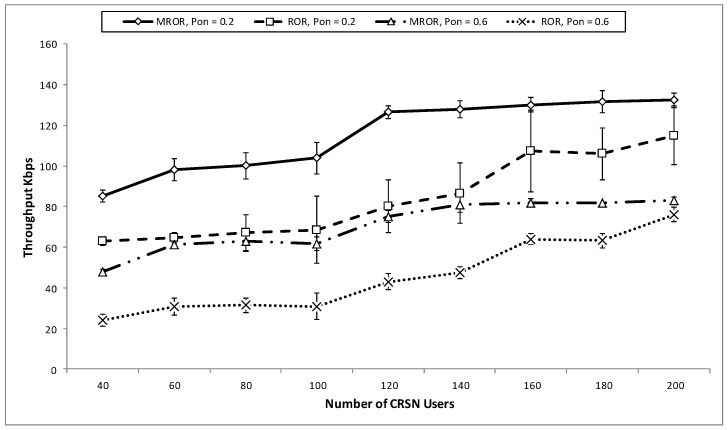
Throughput *vs*. number of network nodes.

**Figure 14:**
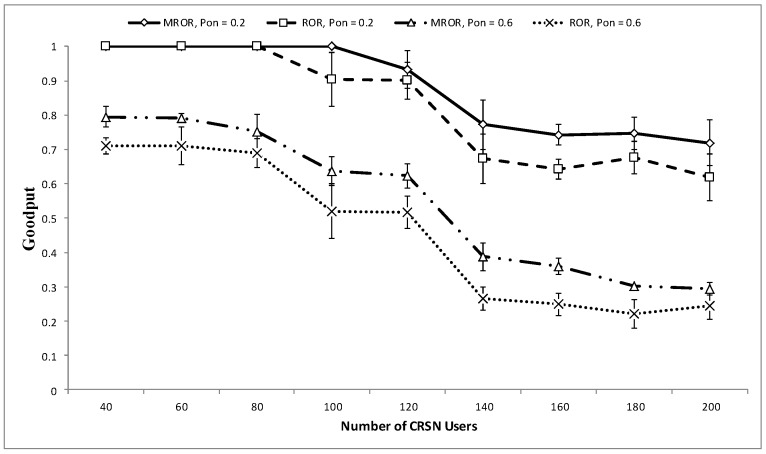
Goodput *vs*. number of network nodes.

In Figure 13 and Figure 14, MROR is compared to ROR to show the effect of utilizing the spatio-temporal opportunities provided by mobility in routing. In both cases, MROR was set at υ=5 m/s, and ROR was set at υ=0 m/s. From Figure 13, it can be observed that in the case of MROR, the throughput initiation increases as the network node density increases. What is responsible for this is that as the number of nodes increases, network traffic emanating from the source increases, and thus, the network at the sink increases. However, the throughput is observed to be somewhat stable when the density is about 120. Likewise, when the PU activity increases, it results in a corresponding decrease in throughput, as channel access time has been reduced. Although the channel access time is reduced, MROR is still able to record a good throughput when it is compared to ROR, which does not consider the spatio-temporal opportunities provided by mobility. It can also be derived that the best operational network density for the operation of MROR is at the point of 120 because of the observed stability of the throughput at this point when compared to ROR. For clearer observance of this property, the goodput performance is presented in Figure 14.

Figure 14 also compares the goodput performance of both MROR and ROR at various PU activities. Although both the network load and the PU adversely affect the final network goodput, it is observed that the PU activity has more effect than the network load. This is because MROR and ROR adopt the same forwarding strategy that considers instantaneous link quality reliability. In addition, the receiver contention prioritization scheme ensures the stability of the goodput as the load increases, since the number of eligible nodes within the priority region also increases as a result. Furthermore, from MROR, it can be also observed that mobility also increases the opportunity for quality links. However, as can also be observed, when the number of contending nodes around the priority regions increases, this also increases the probability of collision, thereby resulting in a decline in the goodput. Unlike in ROR, where the goodput remains stable at intervals within certain SU density levels, a continuous decline is observed in the case of MROR from an SU density level of 120 as a result of the effect of mobility and frequent changes of the position of the nodes with respect to the spatio-temporal characteristic. This is why this density level has been chosen as the best operating value for MROR.

## 7. MROR Comparison

In this section, MROR has been compared to both CRP [11] and ROR. Why CRP [11] was chosen was because it supports the building of routes across the PU coverage area, which is more in line with the CRSN scenario considered in this work. This contrasts with SEARCH [12] and TIGHT [14], which build routes outside the PU coverage area in all cases. In addition, all available protocols have not considered the unique resource-constrained nature of CRSNs. Thus, for this evaluation, CRP [11] is identified as the most appropriate. ROR [13] was used to show the effect of not considering the speed of mobile nodes in the design of on-demand protocols.

**Figure 15:**
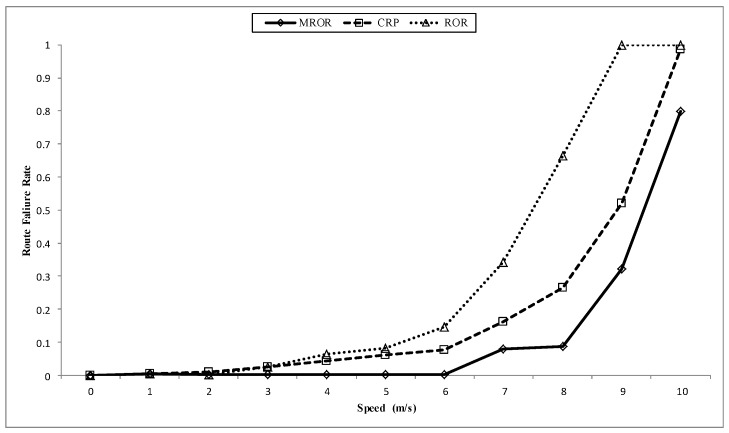
Effect of speed on route stability in cognitive radio sensor network (CRSN).

In Figure 15, the effect of speed on route stability is presented. All protocols can still maintain route stability until speeds of about 2 m/s. Furthermore, because LMR nodes in ROR were originally designed to be static, the frequent recall of the route maintenance module when mobility was introduced made the whole routing scheme become unstable. In the case of CRP, its proactive nature of discovering a new path in case the current route fails is responsible for it maintaining network stability better than ROR. Furthermore, it was observed that as the speed increased, the active route is already expired before the proactive function finishes implementing the choice of alternative route. However, MROR surpasses both CRP and ROR, first because of its VMH zoning system and its forwarding strategy that does not restrict the forwarding node to a single node, as is the case in CRP. This quality makes MROR more robust at handling the issues mobility poses. As a result, a decrease in the failure rate of about 65% is observed by employing the MROR strategy.

**Figure 16:**
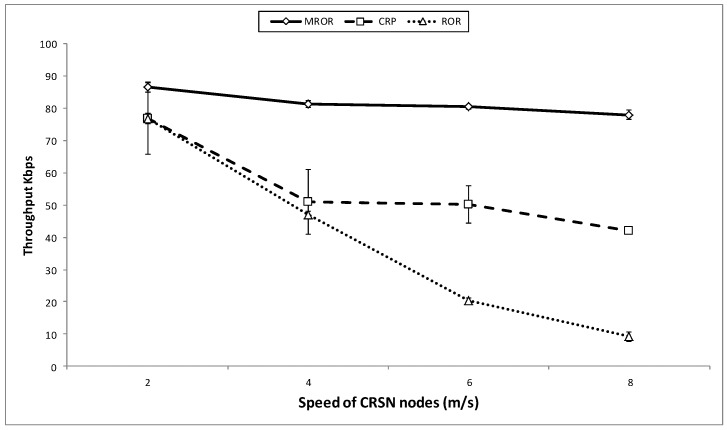
Throughput *vs*. speed of the mobile node.

The effect of the speed on the throughput is illustrated in Figure 16. The route instability of both CRP and ROR definitely affects the throughput as illustrated. However, an additional factor responsible for the observed performance of MROR as compared to the other two is the spatio-temporal property it considers in the route selection stage that greatly improves the throughput at the sink. As in the case of both CRP and ROR, this metric was not considered for route formation. In general, while MROR tries to maintain the throughput as the speed increases, the values for throughput dwindle at increased speed in the case of CRP, and a more drastic decline is observed for the case of ROR.

**Figure 17:**
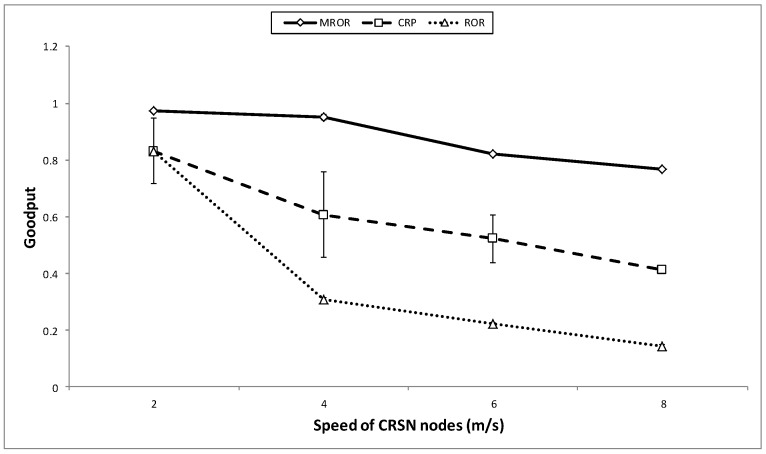
Goodput *vs*. speed of the mobile node.

In Figure 17, the goodput performance of all three cases is illustrated. In general, the speed affects the goodput in all cases, but with varying magnitudes. This effect is most obvious in the case of ROR than CRP, mainly because of the instability of the network at such speeds. However, in the case of MROR, the VMH system greatly enhanced the uninterrupted implementation of the SNR reception metric. In addition, enforcing prioritization for potential receivers also helped the reduction in contention, which subsequently reduced collision during forwarding the packet towards the sink. On the contrary, in the case of CRP, in addition to the instability, the absence of instantaneous channel information often demands the need for multiple re-tries in transmitting the packet. This has also contributed to the observed decline in goodput.

**Figure 18:**
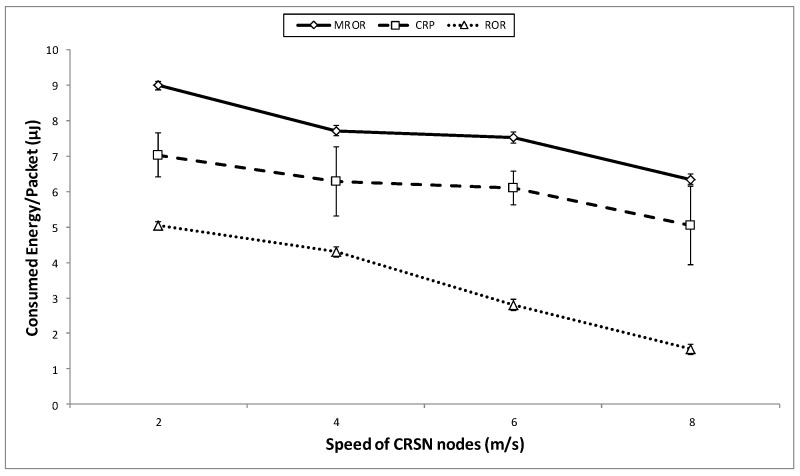
Consumed energy/packet *vs*. speed of the mobile node.

The energy consumed per packet is illustrated in Figure 18. MROR outperforms CRP because MROR is designed for CRSNs; Thus, energy was always considered in the creation of routes and in the forwarding of packets. The forwarding scheme also helps in network load distribution, which also affects the energy of the whole network. In contrast, CRP was not designed for CRSNs; therefore, energy was not considered in either the route election or forwarding stages. In addition, the frequent route request that is initiated by the route instability also contributes to the inefficiency (in terms of energy) when compared to MROR. Another factor responsible for the energy efficiency of MROR is the utilization of idle listening to make critical network decisions, which was not considered in CRP. Furthermore, the extension in hop length offered by MROR has also helped in conserving energy by the reduction of hops to the sink. As in the case of ROR, the main reason that can be attributed to the energy inefficiency is the frequent initiation of the route request and route maintainability that were caused by the speed introduced into the network.

## 8. Conclusions

This paper presented the MROR routing protocol, which addresses the issues of route stability and the spatio-temporal spectrum availability metric in mobile CRSNs. MROR, which was built upon ROR, also ensures the reliability of communication links in CRSN. It was established that typical routes for CRSNs must not always be built away from the PU coverage area, as a target area of interest might lie within a PU coverage area. Thus, typical CRSN mobile routing should jointly create efficient routes within and outside the PU coverage area as the case may be. For routes built outside the PU coverage area, it was observed that the traditional keep-out radius does not properly account for the protection of the PU when mobility is considered. Therefore, a mobility-induced guard (mguard) distance was introduced into the scheme to both ensure proper PU protection and improve the throughput of the mobile nodes. It was also observed that at higher speeds, the sensing interval was more dependent on the speed of the mobile nodes than it was on the statistics of the PU traffic, which is the strategy used by the reviewed works. Thus, the routing metric in MROR both considers the speed and the PU traffic in creating routes. The main metric that makes the speed important in this respect is the spatio-temporal spectrum availability that is mobility induced. The effect of not considering the speed results in a high level of route instability. As a solution, in addition to creating virtual clusters around potential next hop nodes of the selected routes, MROR also introduces a unique hand-over region that helps to greatly reduce the re-initiation of a new route request and route maintenance operations that is usually caused by mobility. As a result, MROR can record a decrease in the route failure rate of about 65% as against other schemes. Furthermore, simulation studies have shown that MROR can improve both the throughput and goodput at the sink in an energy-efficient manner that is required in CRSNs as against reviewed works. It is important to mention that the presented result for the MROR scheme is limited to the smooth mobility model utilized in the study. In addition, the considered PU and SU scenario for this work is static PUs and mobile SUs. For further improvement, we suggest the modification of the scheme to be adaptable to the random waypoint model, while other scenarios in which both PU and SU are mobile should be considered. It is envisaged that this work will be extended by incorporating a suitable transport layer to ensure advanced congestion control to preserve scarce network resources while considering application-based QoS requirements.
